# Neutrophil to lymphocyte ratio predicts bowel ischemia in non-strangulated adhesive small bowel occlusions: a retrospective analysis from an acute care surgical service

**DOI:** 10.1186/s12893-024-02476-2

**Published:** 2024-06-12

**Authors:** Alberto Friziero, Eugenia Rosso, Irene Sole Zuin, Lorenzo Vallese, Simone Serafini, Alessandra Amico, Valeria Valli, Chiara Da Re, Nicola Baldan, Michele Valmasoni, Gianfranco Da Dalt, Cosimo Sperti

**Affiliations:** 1 Department of Surgery, Oncology and Gastroenterology, 1st Surgical Clinic, University of Padua, Via Giustiniani 2, Padua, 35128 Italy; 2 Department of Surgery, Oncology and Gastroenterology, 2nd Surgical Clinic, University of Padua, Via Giustiniani 2, Padua, 35128 Italy

**Keywords:** ASBO, Bowel ischemia, Neutrophil-lymphocyte ratio, Acute care surgery

## Abstract

**Background:**

Adhesive small bowel obstruction (ASBO) is a leading cause of hospitalization in emergency surgery. The occurrence of bowel ischemia significantly increases the morbidity and mortality rates associated with this condition. Current clinical, biochemical and radiological parameters have poor predictive value for bowel ischemia. This study is designed to ascertain predictive elements for the progression to bowel ischemia in patients diagnosed with non-strangulated ASBO who are initially managed through conservative therapeutic approaches.

**Methods:**

The study was based on the previously collected medical records of 128 patients admitted to the Department of Acute Care Surgery of Padua General Hospital, from August 2020 to April 2023, with a diagnosis of non-strangulated adhesive small bowel obstruction, who were then operated for failure of conservative treatment. The presence or absence of bowel ischemia was used to distinguish the two populations. Clinical, biochemical and radiological data were used to verify whether there is a correlation with the detection of bowel ischemia.

**Results:**

We found that a Neutrophil-Lymphocyte ratio (NLR) > 6.8 (OR 2.9; 95% CI 1.41–6.21), the presence of mesenteric haziness (OR 2.56; 95% CI 1.11–5.88), decreased wall enhancement (OR 4.3; 95% CI 3.34–10.9) and free abdominal fluid (OR 2.64; 95% CI 1.08–6.16) were significantly associated with bowel ischemia at univariate analysis. At the multivariate logistic regression analysis, only NLR > 6.8 (OR 5.9; 95% CI 2.2–18.6) remained independent predictive factor for small bowel ischemia in non-strangulated adhesive small bowel obstruction, with 78% sensitivity and 65% specificity.

**Conclusions:**

NLR is a straightforward and reproducible parameter to predict bowel ischemia in cases of non-strangulated adhesive small bowel obstruction. Employing NLR during reevaluation of patients with this condition, who were initially treated conservatively, can help the acute care surgeons in the early prediction of bowel ischemia onset.

**Supplementary Information:**

The online version contains supplementary material available at 10.1186/s12893-024-02476-2.

## Background

Adhesive Small Bowel Obstruction (ASBO) is the most common cause of intestinal obstruction and a leading cause of morbidity in emergency surgery [1–4]. With nearly 5% of patients experiencing this pathology after abdominal or pelvic surgery, ASBO represents a significant burden on the healthcare system. According to Bologna Guidelines [5], the initial evaluation consists of clinical history, physical examination, laboratory tests and abdominal Computed Tomography (CT scan) with contrast [6, 7].

Currently, conservative treatment is accepted as primary approach for managing patients without evidence of strangulation and this strategy can lead to a complete resolution in approximately 70% of cases. For the remaining 30%, surgical intervention becomes imperative due to the failure of non-operative management. Among the surgically treated patients, approximately one-third exhibit intraoperative findings of bowel ischemia, resulting from the progression of the obstruction. It is well established that bowel ischemia due to adhesion follows a time-dependent course, that can start as a reversible condition and then develop into necrosis and possibly subsequent intestinal perforation. Due to the diagnostic challenges and the alarming consequences of delayed treatment of bowel ischemia, in recent literature, many attempts were made to discern the presence of bowel ischemia from simple bowel obstruction [8]. Several parameters, including fever, leukocytosis, elevated lactate levels, localized abdominal pain, have been associated with the likely presence of bowel ischemia but no factor was considered statistically relevant [9]. Although Computer Tomography (CT) can assess specific signs of bowel ischemia in non-strangulated adhesive small bowel obstruction, none of these findings provide high predictive value for the detection of this condition [10, 11]. Given the high incidence of ASBO and the necessity of its early management, discovering other predictive factors becomes mandatory to identify additional predictive factors to discern patients for whom initial conservative treatment is likely to be unsuccessful.

Neutrophil-Lymphocyte Ratio (NLR) and Platelet-Lymphocyte Ratio (PLR) are serum biomarkers calculated dividing the absolute neutrophil counts (NLR) or absolute platelet counts (PLR) by the absolute lymphocyte counts. Several studies tried to associate these biomarkers ratio with the presence of ischemia in different clinical situations [12–14]. However, their use as predictors of bowel ischemia in ASBO is very limited.

Our studies aim to assess the value of clinical, biochemical (including NLR and PLR) and radiological parameters in predicting the presence of bowel ischemia in non-strangulated adhesive small bowel obstruction.

## Materials and methods

Medical records of 128 patients, operated at Department of Acute Care Surgery of Padua General Hospital with an admission diagnosis of non-strangulated adhesive small bowel occlusion between August 2020 and April 2023, were retrospectively collected. Indication for surgery was decided by acute care surgeons for patients who failed initial conservative treatment, according to Bologna Guidelines [5]. Patients who required emergent surgery for free perforations or bowel strangulation were purposely excluded, as well as hematological and immunocompromised patients. Other causes of small bowel obstruction except adhesions (i.e. cancer or bezoars) were also excluded. Patients were categorized into two groups: one consisted of patients with intraoperative findings of bowel ischemia (Ischemic group) and one consisted of patients without evidence of bowel ischemia/infarction at the time of surgery (Control group). The following demographic and clinical-pathological variables were analyzed: (i) age, (ii) gender, (iii) ASA score, (iv) Charlson comorbidity index, (v) presence of Systemic Inflammatory Response Syndrome, (vi) time from onset of occlusive symptoms, (vii) number of previous abdominal operations, (viii) interval time between admission to operating room. All post operative complications and reoperations that occurred during hospitalization were registered and graded according to the Clavien-Dindo classification [15].

Preoperative biochemical parameters were recorded for analysis: (i) White blood cell (WBC) count, (ii) neutrophil count, (iii) lymphocyte count, (iv) hemoglobin level, (v) platelets count, (vi) C-Reactive Protein and (vii) lactate levels. Neutrophil to lymphocyte (NLR) and platelets to lymphocyte ratio (PLR) were calculated to evaluate whether there is a correlation with intestinal ischemia. For each patient we considered the latest blood sample taken before the surgical operation (all samples were collected within 12 h before surgery).

At admission, all patients underwent abdominopelvic Multidetector Computed Tomography (MDTC) involving pre and portal phase CT scans. All imaging were reviewed by an expert radiologist with ten years of experience in emergency abdominal radiology. Computed tomography findings included: (i) intestinal pneumatosis, defined as the presence of gas in the bowel wall; (ii) decreased bowel wall enhancement, defined as a decrease in the enhancement of dilated bowel compared with normal bowel wall, (iii) more than 3 mm bowel wall thickening; (iv) transition zone defined as the specific zone in which there is an exchange between the caliber of the dilated proximal loops and that of collapsed distal loops of the bowel; (v) mesenteric haziness defined as increased fat attenuation in the mesentery of the dilated bowel; (vi) whirl signs defined as a spin of the mesenteric vessels and fat with rotated bowel loop; (vii) free peritoneal fluid defined as presence of ascites in the peritoneal cavity.

Presence of volvulus and closed-loop obstruction were excluded from analysis because these conditions are highly at risk of strangulation, therefore these patients were urgently operated on [16].

## Statistical analysis

Demographics, clinicopathological, biochemical and radiological parameters were analyzed using numbers and proportions or medians with interquartile ranges (IQR), when appropriate. Categorical variables were compared using Chi [2] or Fisher exact test as appropriate, whereas continuous variables were compared with Mann-Whitney test. Cut-off value for NLR was calculated from Receiver Operating Characteristic (ROCs) curve analysis. The optimal cut-off was identified as the nearest point of intersection to the top left-hand corner, between the ROC curve and a diagonal line drawn from the top right-hand corner to the bottom left-hand corner of the graph. Multivariate logistic regression analysis was performed to identify preoperative factors independently associated with intestinal ischemia. This model was constructed combining all the preoperative variables that were significant at univariate analysis.

All statistical analyses were carried out using Graph Pad Prism 9 (GraphPad Software Inc. California, USA) and a *p* < 0.05 was considered significant.

This retrospective study was approved by the local institutional review board and followed local protocols, in accordance with the Declaration of Helsinki.

## Results

A total of 403 patients were admitted to our department with a diagnosis of adhesive small bowel obstruction. Among them, forty-two patients (10.4%) were diagnosed with free intestinal perforation or overt intestinal strangulation and required urgent surgical intervention. For the remaining 361 patients, we started a non-operative approach with *nil per os*, intravenous fluid administration and nasogastric tube placement, in accordance with Bologna guidelines. This approach resulted in complete resolution in 233 cases (65%), whereas 128 patients (35%) underwent surgery for failure of non-operative treatment, subsequently becoming subjects of our analysis. The primary indications for surgical exploration (Table 1) were exacerbation of abdominal pain (43%) and elevation of biochemical inflammation parameters (28%).

**Table 1:** Clinical-pathological reasons for failure of conservative treatment in the study group

Clinical-pathological variables	Entire cohort (*N* = 128)
Exacerbation of abdominal pain, n (%)	55 (43%)
Elevation of inflammatory biomarkers, n (%)	36 (28%)
Persistent obstruction > 72 h, n (%)	5 (4%)
Increased NGT drainage, n (%)	10 (8%)
Failure of water-soluble contrast administration, n (%)	5 (4%)
Peritonitis, n (%)	17 (13%)

*Abbreviations* NGT Nasogastric tube

Out of the 128 patients operated for ASBO, 41 (32%) were found to have intraoperative evidence of bowel ischemia: in six cases (15%), ischemia was resolved after hand-lysis and irrigations of suffering bowel loop with warm saline solution, whereas for 35 patients (85%) a small bowel resection for irreversible bowel ischemia was necessary.

Summary STARD 2015 flow diagram can be found in Supplementary Fig. 1.

Pathology of the resected specimen revealed full thickness ischemia in 29 cases (83%) and transmural hemorrhage, congestion and ulceration in 6 cases (17%). The remaining eighty-seven (68%) patients presented bowel obstruction without signs of ischemia and were treated only with adhesion-lysis or hand-lysis.

Surgical details and post operative outcomes are reported in Supplementary Table 1. Laparoscopy was performed successfully in 40% of cases, and when a bowel resection was necessary, an extracorporeally isoperistaltic anastomosis was performed through a periumbilical incision. Open conversion through a midline laparotomy was necessary in other 21 patients (16%), mostly in the Ischemic group (p 0.04). Analysis of surgical outcomes revealed that patients of the Ischemic group presented a higher rate of postoperative morbidity and were more frequently admitted to intensive care units (p 0.001). Post operative mortality was 4.8% in the Ischemic group, with one patient who died of septic shock and one of acute myocardial infarction. No mortality was reported in the Control group.

Demographics, clinical and preoperative biochemical variables and CT findings of the two groups are reported in Table 2. Median age of the entire cohort was 73.5 (IQR 62–81) years and male sex was predominant (52%). When comparing American Society of Anesthesiologists (ASA) functional status in the two groups, the Ischemic group shows a significant majority of patients with ASA score of 3 or higher (p 0.02). In contrast, there was no statistically significant difference concerning the Charlson Comorbidity Index and the presence of Systemic Inflammatory Response Syndrome (SIRS) at presentation. Median time from admission to the operating room was of 17 (IQR 9–48) hours in the Control group, slightly higher compared to the 12 (8–26) hours for patients in the Ischemic Group.

**Table 2:** Demographic information, preoperative biochemical and radiological variables

Variables	Entire cohort *N* 128	Control group *N* 87	Ischemic group *N* 41	*P* value
Sex, *n* (%)				
Male	65 (51%)	47 (54%)	18 (43%)	Ns
Female	63 (49%)	40 (46%)	23 (57%)	
Age, median (IQR)	73.5 (62.2–81)	71 (58–81)	77 (68–81)	Ns
ASA score *≥* 3, n (%)	66 (52%)	38 (44%)	28 (68%)	0.02
Charlson Comorbidity Index, median (IQR)	5 (2–8)	5 (3–7)	6 (3–8)	Ns
Systemic Inflammatory Response Syndrome, n (%)	14 (11%)	9 (11%)	5 (12%)	Ns
Time from onset of occlusive symptoms, days, median (IQR)	1 (1–3)	1 (1–3)	1 (1-3.7)	Ns
Prior abdominal surgery, median (IQR)	1 (1–2)	1 (1–2)	1 (1–2)	Ns
Abdominal quadrants with pain involvement, median (IQR)	3 (1–7)	2 (1–4)	3 (2–7)	Ns
Duration of symptoms before admission, hours, median (IQR)	13 (8–23)	12 (8–21)	15 (8–25)	Ns
Time from admission to operating room, hours, median (IQR)	15 (9–36)	17 (9–48)	12 (8–26)	Ns
Timing of blood samples before surgery, hours, median (IQR)	9 (6-10.5)	10 (6–11)	7 (5-9.5)	Ns
White blood cells count, x10^9, median (IQR)	10.1 (6.7–13.8)	9.5 (6.7–13.1)	11.3 (8.2–16.7)	Ns
Neutrophils count, x10^9, median (IQR)	7.7 (4.8–11.5)	7.4 (4.6–10.8)	8.7 (5.1–11.9)	Ns
Lymphocytes count, x10^9, median (IQR)	1.1 (0.7–1.4)	1.3 (0.9–1.9)	1.1 (0.7–1.3)	Ns
Hemoglobin, g/dL, median (IQR)	13.8 (12.7–15.4)	14 (12.8–15.3)	13.7 (12.3–15.6)	Ns
Platelets count, x10^9, median (IQR)	238 (188–292)	231 (199–281)	259 (164–310)	Ns
C-reactive protein, median (IQR)	15.5 (4.5–58)	11.9 (4.5–51.4)	31 (4-125)	Ns
Lactate, median (IQR)	1.4 (1-2.5)	1.2 (1-2.1)	2 (1.1–3.3)	Ns
NLR, median (IQR)	6.6 (3.9–10.9)	5.6 (3.6–8.8)	10.6 (6.6–13.3)	0.002
PLR, median (IQR)	202 (140–268)	196 (136.4-265.3)	216 (160–280)	Ns
Bowel wall thickening > 3 mm, yes, n (%)	30 (23%)	18 (21%)	12 (29%)	Ns
Transition zone, yes, n (%)	17 (13%)	10 (11%)	7 (17%)	Ns
Pneumatosis, yes, n (%)	10 (8%)	6 (7%)	4 (10%)	Ns
Mesenteric haziness, yes, n (%)	31 (24%)	16 (18%)	15 (37%)	0.03
Whirl signs, yes, n (%)	18 (14%)	11(12%)	7 (17%)	Ns
Decreased bowel wall enhancement, yes, n (%)	10 (8%)	3 (3%)	7 (17%)	0.002
Free fluid, yes, n (%)	86 (67%)	53 (61%)	33 (80%)	0.03

*Abbreviations ASA American society of anesthesiologist; NLR neutrophil-lymphocyte ratio; PLR platelet-lymphocyte ratio; LCR leucocyte-C-reactive protein ratio*

Whilst neither neutrophil nor lymphocyte counts differed significantly between the two groups, the Ischemic Group had significantly higher preoperative NLR (p 0.002). Conversely, PLR didn’t show any difference and therefore it was no longer investigated.

The CT findings of decreased bowel wall enhancement (p 0.002), mesenteric haziness (p 0.03) and free fluid (p 0.03) were more frequent in the Ischemic group. No significant differences were detected in terms of bowel wall thickening, intestinal pneumatosis, presence of transition zone and whirl signs.

**Fig. 1:**
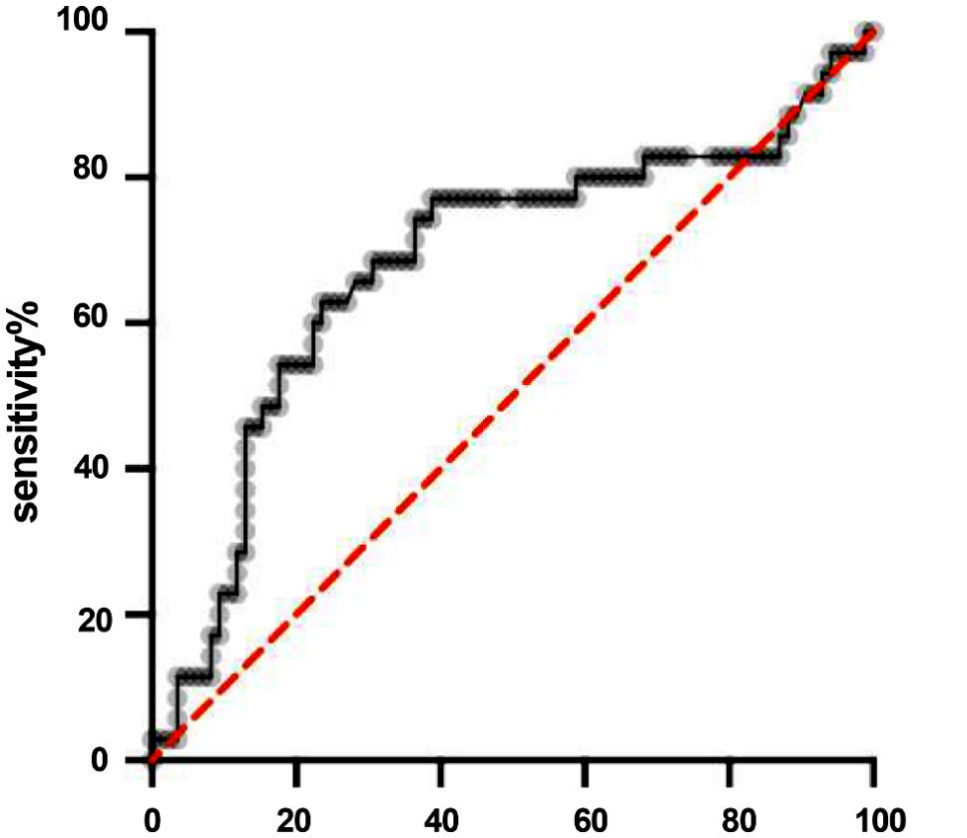
ROC curve analysis for the determination of the best cutoff of Neutrophil to Lymphocyte ratio. The cutoff value was 6.8 with a sensitivity of 78% and specificity of 65% (area under the curve 0.7)

ROC analysis was performed for NLR (Fig. 1) in order to determine the cut off value predictive of intestinal ischemia before surgery. A NLR cut off of 6.8 (AUC 0.7) was found to be associated with small bowel ischemia, with 78% sensitivity and 65% specificity.

The univariate and multivariate analyses of predictive factors for bowel ischemia in adhesive small bowel obstruction are shown in Table 3. There was a statistically significant association between bowel ischemia and NLR > 6.8 (OR 2.9; 95% CI 1.41–6.21), mesenteric haziness (OR 2.56; 95% CI 1.11–5.88), decreased wall enhancement (OR 4.3; 95% CI 3.34–10.9) and free fluid (OR 2.64; 95% CI 1.08–6.16). All these variables were then included in multivariate analysis, showing that only NLR > 6.8 (OR 5.9; 95% CI 2.2–18.6) remained independent predictive factor for small bowel ischemia in non-strangulated adhesive small bowel obstruction.

**Table 3:** Univariate^a^ and multivariate^b^ analysis of preoperative factors associated with intestinal ischemia in adhesive small bowel obstruction

	Ischemia *n* (%)	OR (95% CI)^a^	*P* value	OR (95% CI)^b^	*P* value
**NLR > 6.8, n (%)**	26 (20.3%)	2.9 (1.41–6.21)	0.004	5.9 (2.20–18.16)	0.007
**Mesenteric haziness, n (%)**	15 (11.7%)	2.56 (1.11–5.88)	0.02	2.16 (0.74–6.33)	Ns
**Decreased bowel wall enhancement, n (%)**	7 (5.4%)	4.3 (3.34–10.9)	0.06	10.7 (0.89–19.6)	Ns
**Free fluid, n (%)**	33 (25.7%)	2.64 (1.08–6.16)	0.02	0.54 (0.17–1.58)	Ns

*Abbreviations* ASA American society of anesthesiologist; NLR neutrophil-lymphocyte ratio; PLR platelet-lymphocyte ratio; LCR leucocyte-C-reactive protein ratio

## Discussion

Adhesive small bowel obstruction represents a frequent clinical entity characterized by high morbidity and mortality, and accounts for 9% of emergency surgical admissions at our department. Epidemiological features of our cohort follow the trend of other series with percentages of failure of conservative treatment of approximately 30% and bowel resections ranging from 6 to 13% [17, 18].

In line with the analysis of Margenthaler et al. [19], patients undergoing resection for intraoperative findings of bowel ischemia, presented a risk of adverse outcomes up to 4 times higher when compared with those treated only with adhesion-lysis, confirming the importance of early diagnosis for this clinical condition.

Grey-zone is still represented by patients initially treated with conservative therapy who can develop intestinal ischemia as progression of occlusion. In our report, we evaluated the CT signs commonly considered early predictors of bowel ischemia. They included decreased bowel wall enhancement, bowel wall thickening < 3 mm, mesenteric haziness, peritoneal free fluid, the whirl sign and presence of transition zone. Also of note is the fact that diagnostic performance varies widely across these signs [20, 21], and none of them individually showed both high sensitivity and specificity in predicting bowel ischemia. Another relevant issue is the proven interobserver variability among radiologists in detecting subtle signs of early bowel ischemia [11]. To limit this bias as much as possible, we chose to involve only patients studied with intravenous contrast CT and imaging revision made by an expert radiologist. Moreover, CT scan is usually performed at admission, in most cases several hours or days prior to an eventual surgical exploration, and its use during re-evaluation of patients with non-strangulated adhesive small bowel obstruction is not routinely performed, as it is reserved for selected patients with rapid impairment of clinical conditions. According to our analysis, decreased bowel wall enhancement, presence of mesenteric haziness and free fluid were associated with the presence of bowel ischemia only at univariate analysis. Similar results come from the study of Cox et al. [10] where the presence of mesenteric haziness (sensitivity 88%, specificity 54%), abdominal free fluid (sensitivity 40%, specificity 93%) and the lack of small bowel fecal signs (sensitivity 77%, specificity 52%) seem to be valid predictors of conservative treatment failure, but the authors deduced that only the concomitant presence of all three signs suggests the need for surgery for high suspicion of irreversible obstruction. Decreased bowel enhancement results are also associated with bowel ischemia in the report of Cox et al. [10] with good sensitivity (90%) but lower specificity (30%). These data clarify the role of CT scan as an important but not exclusive exam in the diagnostic workup of ASBO. Therefore, further easy and reproducible tests are needed in order to facilitate the decision-making process and minimize delayed operations.

In our study, at multivariate analysis, only NLR > 6.8 resulted as an independent predictor of intestinal ischemia in non-strangulated adhesive small bowel obstructions. In the English literature, a correlation between an increase in NLR and the risk of bowel ischemia in ASBO was reported by Woodford et al. [12]. Similarly to our study, they discovered that a preoperative NLR of 7.4, measured from the latest blood tests taken, was associated with 85.2% sensitivity (95% CI 71.8–98.6) and 60.3% specificity (95%CI 49.1–71.5) for bowel ischemia in non-strangulated adhesive small bowel obstruction.

An explanation for this difference in cut-offs could be attributed to the fact that we utilized samples collected within 12 h prior to surgery, whereas in the study conducted by Woodford et al., they evaluated samples obtained immediately preoperatively. An increased NLR has also been associated with the severity of other acute care conditions characterized by intra-abdominal inflammatory process or by tissue damage such as trauma, mesenteric ischemia and strangulated inguinal hernia [13, 22–24]. A possible explanation for its relevant role in this setting is that NLR is a biomarker which conjugates two sides of the immune system: the innate immune response mainly due to neutrophils and the adaptive immunity supported by lymphocytes. Conditions characterized by tissue degeneration, such as bowel ischemia, activate the systemic inflammatory response (SIRS) that, in the early phase, consists in neutrophil recruitment, suppression of neutrophil apoptosis and stress-hormone release (i.e. cortisol and catecholamines) [25, 26]. At the base of the NLR functioning, there is the fact that if the body produces more stress hormones, neutrophil levels raise while lymphocyte levels lower. Thus, NLR is often determined by an increase in neutrophils and a decline in lymphocytes. An increase in NLR precedes WBC and CRP alterations, being the first sign of the activation of the immune system during systemic inflammatory response syndrome [27]. While the NLR role is well established, its cut off value in baseline and inflammatory conditions is still under investigation. Forget et al. [28], in a large retrospective case control study, observed that normal NLR in the adult population ranges from 0.78 to 3.53, whereas in the Rotterdam study [29], it was observed that mean NLR in the general population was 1.76 (0.83–3.92). In inflammatory conditions, the cut off varies depending on the severity of the disease. In a recent review, Buonacera et al. [27] proposed the most reliable NLR cut off value for septic patients, based on the extension of the infective process: for local infection the cut-off ranges from 5 to 10, for systemic infection it ranges from 10 to 13, for sepsis varies from 13 to 15, and for septic shock this value exceeds 15. This evidence supports our results, with a NLR cut off (6.8) as early predictor of bowel ischemia, making a disease process still confined at bowel loop damage from obstruction with no systemic repercussions.

The retrospective nature of this study and the relatively small population sample are the main shortcomings of this study. Although the study was conducted in a tertiary hospital, it is a single center study and our results do not allow us to create a reliable prediction model. Despite these limitations, we achieved a slightly lower sensitivity than the only one previously reported in the literature [12], but a higher specificity. Therefore, we believe that our study may provide valuable insights to advance research on this topic and to encourage a larger application of the NLR, particularly during reevaluation of patients with non-strangulated adhesive small bowel obstruction and CT signs of possible early bowel ischemia initially treated with conservative treatment.

In conclusion, adhesive small bowel obstruction (ASBO) represents a common challenge for acute care surgeons with post operative course characterized by high morbidity, in particular concerning patients with intraoperative findings of bowel ischemia. The Neutrophil-to-Lymphocyte Ratio (NLR) serves as a cost-effective, straightforward, reproducible and easily available parameter of stress, inflammation and tissue damage. This finding holds potential significance in identifying high-risk patients who may benefit from surgical exploration in order to minimize delayed operations and reduce both rate and extension of intestinal resections.

### Electronic supplementary material

Below is the link to the electronic supplementary material.

Supplementary Material 1

Supplementary Material 2

## Data Availability

Data collection forms and data used for analysis are available on request, writing an e-mail to the corresponding author (cosimo.sperti@unipd.it).

## Abbreviations

ASA: American society of anesthesiology
ASBO: Adhesive small bowel obstruction
IQR: Interquartile range
MDCT: Multidetector computed tomography
NLR: Neutrophil-Lymphocyte Ratio
CRP: C-reactive protein
PLR: Platelet-Lymphocyte Ratio
ROC: receiver Operating Characteristic
SIRS: Systemic Inflammatory Response Syndrome
WBC: White blood cells
