# Supplementary material for: Neutrophil to lymphocyte ratio predicts bowel ischemia in non-strangulated adhesive small bowel occlusions: a retrospective analysis from an acute care surgical service
Source: BMC Surg. 2024 Jun 12;24:179. doi: 10.1186/s12893-024-02476-2 (PMC11167870; doi:10.1186/s12893-024-02476-2)
Supplement: Supplementary file 1 — Supplementary Material 1 [file 12893_2024_2476_MOESM1_ESM.docx]

| **Variables** | **Entire cohort**  **N 128** | **Control group**  **N 87** | **Ischemic group N 41** | **P value** |
| --- | --- | --- | --- | --- |
| **Laparoscopy, n (%)** | 53 (41%) | 42 (48%) | 11 (26%) | 0.02 |
| **Conversion to laparotomy, n (%)** | 21 (16%) | 9 (10%) | 12 (29%) | 0.04 |
| **ICU admission, n (%)** | 33 (26%) | 15 (17%) | 18 (44%) | 0.001 |
| **Overall morbidity, n (%)** | 36 (28%) | 18 (20%) | 18 (43%) | 0.004 |
| **Major morbidity, n (%)** | 7 (5%) | 2 (2.2%) | 5 (12%) | 0.03 |
| **Mortality, n (%)** | 2 (1.5%) | 0 (0%) | 2 (4.8%) | Ns |
| **Length of stay, day, median** | 7.5 (5-10) | 7 (5-8.5) | 9 (6-12) | Ns |

***Supplementary Table 1:*** *Surgical details and post operative course of the study group. Overall morbidity includes patients with post operative course categorized as Clavien Dindo >1. Major morbidity includes patients with post operative course categorized as Claviend Dindo >3b*.

*Abbreviations: ICU Intensive Care Unit.*
