# Supplementary material for: Neutrophil to lymphocyte ratio predicts bowel ischemia in non-strangulated adhesive small bowel occlusions: a retrospective analysis from an acute care surgical service
Source: BMC Surg. 2024 Jun 12;24:179. doi: 10.1186/s12893-024-02476-2 (PMC11167870; doi:10.1186/s12893-024-02476-2)
Supplement: Supplementary file 2 — Supplementary Material 2 [file 12893_2024_2476_MOESM2_ESM.docx]

**Supplementary figure 1:** Summary STARD 2015 flow diagram of study population and management.
